# Determination of 5‐Hydroxymethylfurfural Content in Marketed Honey: A Modified RP‐HPLC Method

**DOI:** 10.1155/ianc/9780039

**Published:** 2026-04-21

**Authors:** Barbara Adu-Brimpong, Abena Amponsaa Brobbey, Joseph Kwasi Adu, John Nii Addotey, Mustapha Kobina Abeka, Isaac Yaw Attah

**Affiliations:** ^1^ School of Pharmacy and Pharmaceutical Sciences, Department of Pharmaceutical Chemistry, University of Cape Coast (UCC), Cape Coast, Ghana, ucc.edu.gh; ^2^ Faculty of Pharmacy and Pharmaceutical Sciences, Department of Pharmaceutical Chemistry, Kwame Nkrumah University of Science and Technology (KNUST), Kumasi, Ghana, knust.edu.gh

**Keywords:** 5-hydroxymethylfurfural, honey, method development, reverse-phase HPLC

## Abstract

Honey is a nutritive sugar alternative, well‐accepted over decades due to its natural source, and a global populace drive toward natural and healthier lifestyle options. Its quality assessment includes 5‐hydroxymethylfurfural (5‐HMF) content, which measures freshness, but has been linked to cancer. The ability to test for this neo‐formed contaminant, 5‐HMF, should therefore not be compromised, even in a resource‐constrained setting. In this study, a modified reversed‐phase HPLC method for the assay of 5‐HMF levels in honey has been developed. A nonpolar C‐18 column of dimension 3.0 × 150 mm; 2.7 μm was the stationary phase of choice, while a combination of 10:90% v/v of methanol and distilled water, with 1% formic acid, was employed as the mobile phase. Detection was by diode array at a wavelength of 295 nm, using an injection volume of 1 μL. At a flow rate of 0.5 mL/min, the total runtime of the method was 8 min, with the average retention time for 5‐HMF recorded at 3.87 ± 0.05 min. Validation of the method was conducted using International Council for Harmonisation guidelines, over the range of 1.25–30.0 μg/mL. It was further employed to quantify 5‐HMF in 20 honey samples on the Ghanaian market with varying origin, extraction source and production state, using methanol as the dissolution solvent to ensure stability of the target compound throughout the analysis. 5‐HMF levels assayed in the honey samples ranged from 0.8176 ± 0.182 to 81.1619 ± 2.1169 mg/kg of honey. At the time of analysis, 5‐HMF contents of all samples except one were within the acceptable limit of ≤ 80 mg/kg of honey, set by the International Honey Commission. The 5‐HMF levels quantified in market samples approved by Ghana’s Food and Drugs Authority were within the acceptable limit. The developed method is comparatively cheaper and can be successfully used to analyse 5‐HMF in honey samples.

## 1. Introduction

Honey is a sweet, viscous natural food product obtained from the activity of honeybees, *Apis mellifera* L. Its major primary building block is the sugary secretions or nectar of plants or secretions of living parts of plants, which the bees collect, transform, store and leave in the honeycomb to ripen and mature [1]. Honey contains important macromolecules and compounds including but not limited to proteins, lactone, antibiotic‐rich inhibin, enzymes, phenols, antioxidants, amino acids, gluconic and organic acids, flavonoids, vitamins and minerals [2]. Its medicinal and pharmaceutical potential is diverse and significant and includes anticancer, antibacterial, antiviral and antiparasitic properties [3].

Its sweetness is from fructose and glucose which are monosaccharides. Pure honey is believed not to favour the growth of most microorganisms; hence, once sealed, it can be stored for thousands of years without spoiling [2].

After extraction, regular honey may be taken through a variety of processing, including pasteurisation. Pasteurisation improves the honey’s appearance and kills yeast cells, thereby extending its shelf‐life but can affect its taste. Though pasteurisation usually makes regular honey smooth and clear, it is believed to reduce the number of antioxidants and nutrients in the honey [4].

**FIGURE 1:**
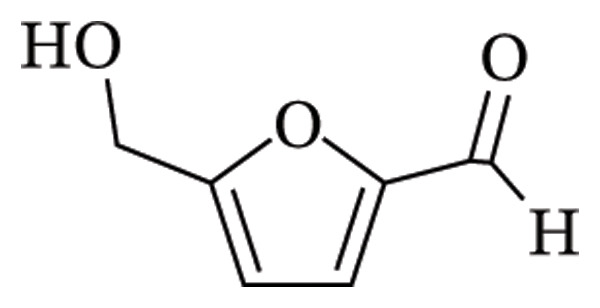
Structure of 5‐hydroxymethylfurfural (5‐HMF).

5‐hydroxymethylfurfural (5‐HMF) is known to be an indicator of stress during food processing, handling and storage [4]. It is one of the neo‐formed compounds formed from heat treatments of carbohydrate‐containing foods by the Maillard reaction. This reaction is a nonenzymatic browning chemical process characterised by acid‐catalysed dehydration of sugars and proceeds faster when heat is applied [5]. Investigations have found that HMF is harmful to human health, causing cytotoxicity to mucous membranes, skin and upper respiratory tract. It has been reported to be mutagenic, causing chromosomal abnormalities and cancer in both humans and animals [6, 7]. The ingestion limit for 5‐HMF is still unclear; its metabolism, biotransformation, excretion, and consequently, clearance rate from the body rely on an individual’s organ function [6]. The chemical structure of 5‐HMF is shown in Figure 1.

Assessing 5‐HMF levels in food products as part of attempts to safeguard food cannot be undermined [8–10].

Different methods have been successfully employed to analyse 5‐HMF levels in various food products such as fruit juices, soft drinks, syrups and honey. Spectrophotometric methods recommended by the International Honey Commission (IHC) for the determination of 5‐HMF in honey are relatively cumbersome, lack specificity and employ some toxic chemicals like p‐toluidine, which is known to be carcinogenic [11, 12]. The development of sensitive and rapid methods for analysing dietary contaminants is continuously evolving, with more automated methods like that developed by Tong et al., 2022, which inculcates advanced extraction procedures for compounds in matrices and hyphenated chromatographic techniques [13].

The official reversed‐phase high‐performance liquid chromatography (RP‐HPLC) method by the IHC, developed for the determination of 5‐HMF in honey, has been in existence since 1980. It required less cost‐effective procedures but takes relatively longer time to complete, hence the development of an alternative method which requires less time [14]. Some reversed‐phase chromatographic methods developed for assaying honey, other food and biological samples either involved multiple procedures to be carried out on samples including extraction as seen in Hardt‐Stremayr et al., 2013, or involved relatively more expensive solvents, like acetonitrile, or a higher percentage of organic solvents like methanol to readily available solvent like distilled water as mobile phases [15–18].

In addition, dissolution solvent of choice, water, utilised in the official IHC method, the comparative study done by Martysiak‐Żurowska & Borowicz, 2009, and the modification by Ünüvar, has been reported to influence the stability of 5‐HMF during analysis [19].

Both the Codex Alimentarius Commission and the European Union established that 5‐HMF concentration in honey after processing shall not be more than 40 mg per kg. However, in the case of honey originating from countries or regions with tropical ambient temperatures and blends of these honeys, the HMF content shall not be more than 80 mg/kg [1, 20]. This study highlights the measurement of the 5‐HMF content of sampled honey using a more economical, alternative HPLC method, which satisfies the requirements of the International Conference on Harmonisation of Technical Requirements for Registration of Pharmaceuticals for Human Use (ICH) [21].

## 2. Materials and Methods

### 2.1. Samples

Twenty honey samples were obtained from April to May 2022, from supermarkets, pharmacies, local honey farmers and hawking vendors, in 12 towns across 7 regions of Ghana, which are popular hotspots where honey is sourced. Collection of samples from the other regions was unsuccessful due to lack of reliable sources at the time of collection. Description of samples is well documented including name, source, type, dates of manufacture and expiry, batch numbers and Food and Drugs Authority registration numbers. Samples with documented expiration dates on the label all had at least 6 months of their shelf lives remaining. They were then given unique codes; all 15 samples of honey produced in Ghana begin with ‘G’, while all 5 foreign brands (from Egypt, South Africa and United Arab Emirates) were denoted ‘F’.

### 2.2. Chemicals and Reagents

Methanol (grade HPLC/UHPLC; assay 100.0%; *ρ* 0.79 kg/L; Lot 20J084008) was obtained from VWR International S. A. S; formic acid (grade analytical reagent; assay 90%; Lot 1405524) was obtained from Fisher Scientific, UK, and freshly distilled water. A reference sample of 5‐HMF was obtained from Sigma‐Aldrich Co., U.S.A (assay: ≥ 99%, CAS 67‐47‐0). It was stored at the recommended temperature of 2°C–8°C, to keep the integrity of the compound over the period of analysis.

### 2.3. Instrumentation

Agilent 1260 Infinity II HPLC (quaternary pump G711B; vial sampler G7129A; multicolumn thermostat G7116A; diode array detector WR G7115A), Poroshell 120 C‐18 column (3.0 × 150 mm; 2.7 μm) and a UV spectrophotometer (Jenway 7513) was utilised.

### 2.4. Preparation of Reference Solutions

Stock solution of concentration 1000.0 and 10.0 μg/mL working concentration of the reference sample of 5‐HMF were prepared using methanol.

Reference solutions were prepared by diluting specific volumes of the stock solution with methanol.

### 2.5. Preparation of Test Solutions

Aliquots of the honey samples were weighed to prepare sample solutions in methanol. The solutions were filtered before HPLC analysis.

### 2.6. Method Development

Developing the method was based ultimately on its ability to identify and quantify all the samples with little to no interference. Chromatographic conditions were varied progressively, using cost‐friendly solvents to achieve the most optimal separation as well as good peak characteristics for the sample. Chromatographic peak resolution was considered as the metric of choice to evaluate the quality of separation [22]. A homogenous mixture of water and methanol was used as the mobile phase for preliminary runs. An ion‐pairing agent, formic acid, was employed to improve the resolution of the chromatograms. Methanol was also used as the diluent for all samples, based on the consistent results and good peak characteristics observed over the period of the development of the method.

### 2.7. Method Validation

#### 2.7.1. Specificity

The working concentrations of 10.0 μg/mL of pure 5‐HMF prepared were analysed with the developed method. Blank solutions were equally subjected to the chromatographic conditions, and the chromatograms generated after multiple determinations were compared.

#### 2.7.2. Linearity and Range

Varying concentrations of the pure sample—1.25, 2.5, 5.0, 10.0, 15.0, 20.0, 25.0 and 30.0 μg/mL—were prepared. Triplicate runs were done for each concentration, and the corresponding peaks areas that resulted were recorded. Subsequently, a graph of the peak area against the corresponding concentrations was plotted to calculate the *R* ^2^ value.

#### 2.7.3. Precision

Repeatability of the analytical method was measured by running the working solution a total of six times. Intermediate precision was then done by analysing the working solutions each day for three different days. The RSDs of the recoveries were also calculated.

#### 2.7.4. Accuracy

Three different concentrations around the working concentration of the pure sample corresponding to 80%, 100% and 120% were prepared. Each concentration was run with the developed method in triplicate, and the average percentage recoveries were calculated.

#### 2.7.5. Robustness

The robustness of the method was determined by varying the column temperature and wavelength of detection. The temperature of the column compartment of the HPLC set‐up was altered from 20°C to 18°C and 22°C, and the wavelength of detection was adjusted to 290 and 297 nm to ascertain the robustness of the method. Corresponding RSDs were calculated from triplicate determinations.

#### 2.7.6. Stability of Solution

Two different concentrations of 1000.0 μg/mL stock solution and 10.0 μg/mL working solutions of the reference sample were prepared. An aliquot of the concentrations prepared was pipetted into separate air‐tight vials and refrigerated. A similar set of aliquots were left on the shelf at room temperature. All aliquots were monitored over a period of about 65 days.

### 2.8. Analysis of Sampled Products

Honey solutions were prepared by dissolving the appropriate mass of each of the honey samples in methanol. These solutions were filtered through syringe filters before analysis. Three determinations were carried out on each sample solution. Percentage content of 5‐HMF in each sample was calculated using peak areas.

### 2.9. Statistical Evaluation of Results

Statistical analysis of the honey samples and the 5‐HMF content between the different brands of honey was performed using *Microsoft Excel* and *GraphPad Prism* 8. The average content of 5‐HMF in milligrams per kilogram of honey, determined using the developed method for the various market samples, was compared using ordinary one‐way ANOVA and unpaired *t*‐test.

## 3. Results and Discussion

### 3.1. Method Development and Validation

The HPLC method for assaying 5‐HMF from honey is considered to be sensitive, automated and able to exclude interference. Further modifications and development have, however, been recommended as reported by Shapla et al. [23].

The stationary phase employed is a silica‐bonded C‐18 column, with dimensions 3.0 × 150 mm and particle size 2.7 μm, taking into consideration the polarity of the compound of interest. The final composition of the mobile phase was 10:90 v/v of methanol:distilled water, with 1% formic acid. This constitution fits the aim of developing an economical method. The best flow rate selected was 0.5 mL/min as it resulted in better retention times and proper resolution of 5‐HMF. Mean retention time of 3.8737 min ± 0.05 observed confirms a good interaction between the compound of interest and the stationary phase. This is remarkable because the run time of the analysis can be performed in about 5 min as compared to the original HPLC method of Jeuring and Kuppers, which had a run time longer than 8 min and other modified methods which have been developed [14, 24].

**FIGURE 2:**
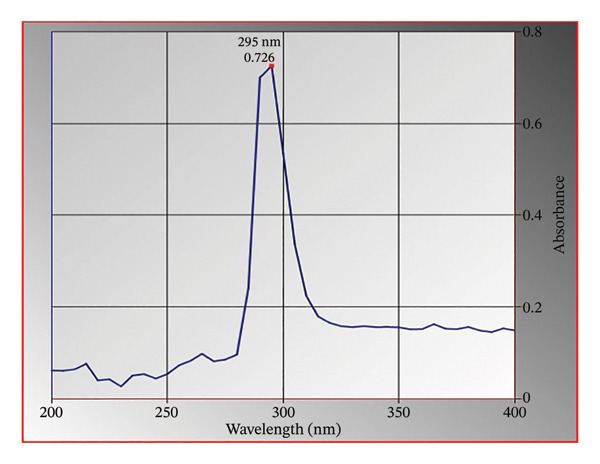
UV spectrum of reference 5‐HMF in methanol. Maximum absorption is observed at 295 nm.

The reference sample absorbed UV radiation considerably from 280 to 310 nm, with maximum absorption observed at 295 nm, after a scan from 200 to 400 nm, as shown in Figure 2; thus, it was chosen as the wavelength of detection.

**FIGURE 3:**
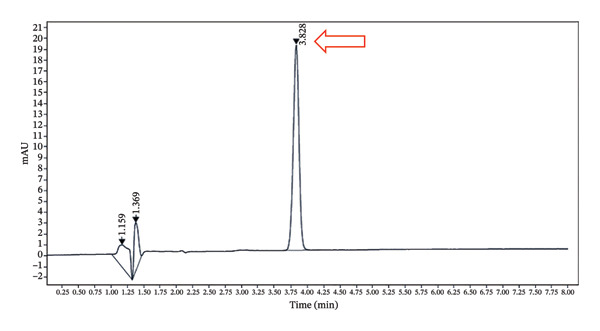
Chromatogram of the 5‐HMF reference sample showing the peak at a retention time of 3.8 min.

**FIGURE 4:**
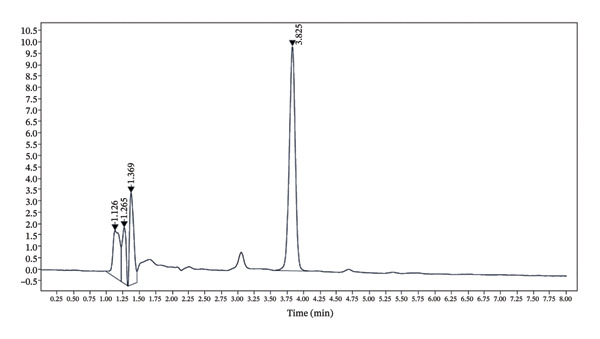
Chromatogram of the honey sample showing the peak of 5‐HMF. Similar peak nature and retention time of 3.8 as in the reference sample may indicate the presence of 5‐HMF.

The developed method was validated based on parameters recommended by the ICH guidelines. The corresponding results are shown in Figures 2, 3 and 4 and Tables 1, 2, 3, 4 and 5.

**TABLE 1:** Results from regression analysis in test for linearity.

Best‐fit values	
Slope	9.5320
y‐intercept	24.414
x‐intercept	−2.578
1/slope	0.1050
*R* ^2^	0.9997
Sy.x	1.438
St. error, slope	0.05069
St. error, y‐intercept	0.8563

*Note:* *p* value < 0.0001, 95% confidence interval.

**TABLE 2:** Results showing repeatability of the developed method.

Expected concentration	Runs	Peak area	Concentration recovered (μg/mL)	% Recovery	Standard deviation
10.00 μg/mL	1	120.679	10.0701	100.701	0.9991
	2	117.986	9.7853	97.853	
	3	119.245	9.9185	99.185	
	4	119.001	9.8927	98.9269	
	5	119.971	9.9952	99.9524	
	6	118.746	9.8657	98.65726	
Av. % recovery				99.2127	RSD = 1.0079%
Acceptance criteria					< 2%

**TABLE 3:** Results of intermediate precision.

Concentration	Day	^∗^Mean recovery	% St. dev.
10.00 μg/mL	1	99.185	
5‐HMF	2	101.174	1.1645
	3	97.854	
	Average	99.0710	RSD = 1.18%
Acceptance criteria			< 2%

^∗^Mean of triplicate determinations.

**TABLE 4:** Results showing accuracy of results from the developed method.

Sample	%	Expected concentration (μg/mL)	^∗^Average peak area	^∗^Mean amount recovered (μg/mL)	Mean % recovery^∗^
5‐HMF	80	8.00	101.478	8.040	100.500 ± 0.730
	100	10.00	119.705	9.967	99.671 ± 0.423
	120	12.00	140.377	12.153	101.273 ± 0.583
Acceptance criteria					98%–102%

^∗^Mean of triplicate determinations.

**TABLE 5:** Robustness of the developed method at altered conditions of temperature and wavelength of detection.

Varied condition	Column temperature (°C)			Wavelength (nm)		
	18	20	22	290	295	297
Av. peak area^∗^	117.896	120.006	120.101	120.182	120.006	116.988
Av. concentration^∗^	9.776	9.999	10.009	10.018	9.999	9.679
% Mean recovery	97.76	99.99	100.09	100.18	99.99	96.79
% RSD	1.33			1.92		

^∗^Mean of triplicate determinations.

The stock and working solutions were seen to be very stable over the study period, as shown in Figure 5, hence preserving the integrity of the target compound during analysis.

FIGURE 5(a) Plot showing the stability of the stock solution employed in the method development (b) plot showing the stability of the working solution employed in the method development.

**Figure:**
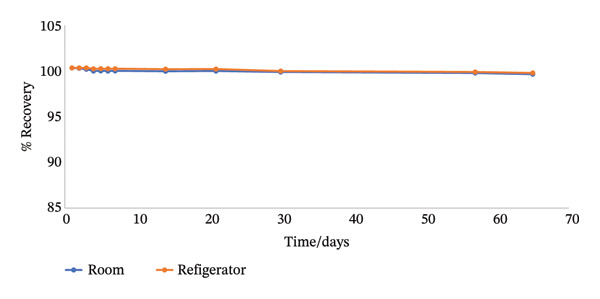
(a)

**Figure:**
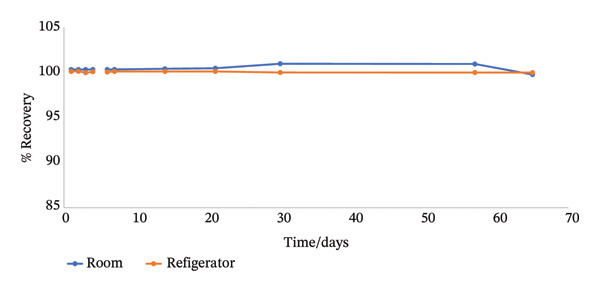
(b)

#### 3.1.1. Specificity

The results of the specificity of the method are shown in Figures 3 and 4.

#### 3.1.2. Linearity and Range

**FIGURE 6:**
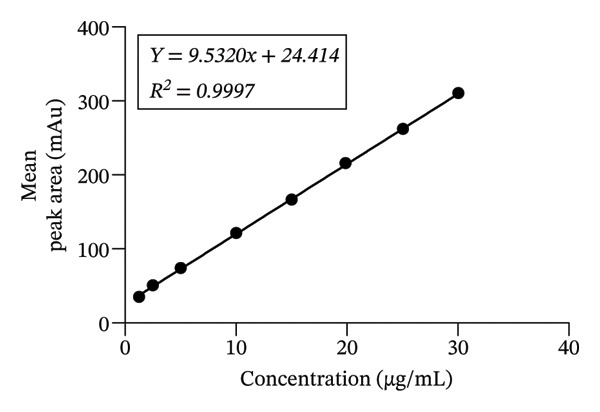
Proof of linearity and its corresponding concentration range for achieving linearity. A plot of the concentrations used (abscissa) and their peak areas ± SEM (ordinate).

The calibration curve for the reference 5‐HMF showed good linearity and range with a high correlation factor (Figure 6).

#### 3.1.3. Precision

Tables 2 and 3 show the precision of the method with multiple runs and the precision of the method on different days.

#### 3.1.4. Accuracy

The accuracy of the method is represented in Table 4.

#### 3.1.5. Robustness

Table 5 shows details of the results by varying two conditions.

#### 3.1.6. Stability of Solutions

The results show that both the stock and working solutions were stable over a period of 65 days under conditions of room temperature as well as refrigeration.

### 3.2. Analysis of Samples

**FIGURE 8:**
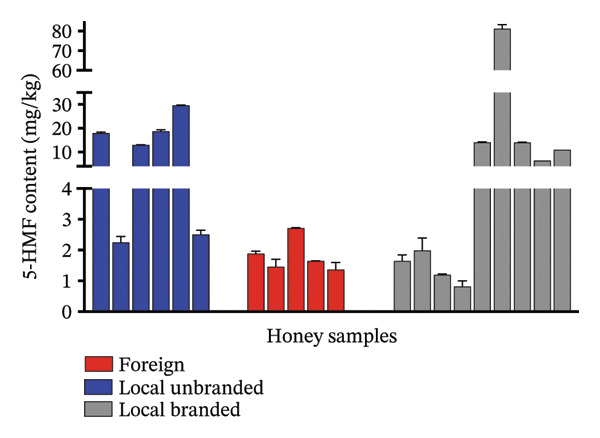
5‐HMF content categorised based on where they were obtained and branding.

**FIGURE 9:**
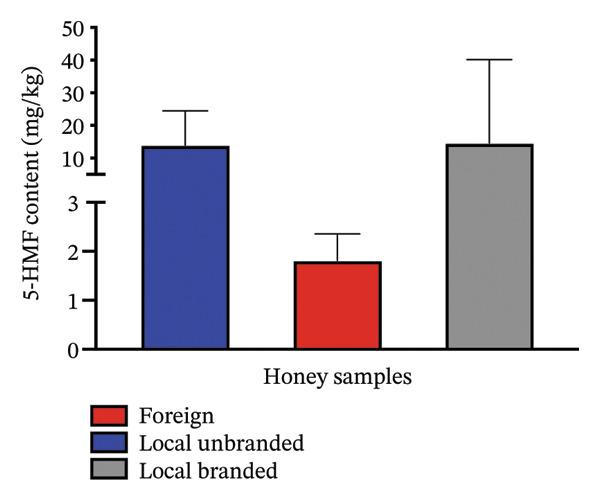
Comparison of the content of 5‐HMF in the analysed honey samples. Results are presented as means ± standard deviation and were analysed using ordinary one‐way ANOVA (*p* value = 0.4330, 95% CI) (grouped mean of local unbranded = 13.99 ± 10.50, foreign = 1.814 ± 0.5412, and local branded = 14.68 ± 25.52).

The characteristics of the market samples of honey, a map of sampling sites, their regional distribution and their respective 5‐HMF content are shown in Figures 7, 8, 9 and 10 and Table 6. There were 4 farmed honey and 9 obtained from the wild, while 7 were of unspecified source (see Figure 1).

FIGURE 7(a) A map of Ghana showing the sampling sites. (b) Distribution of the 15 honey samples obtained in Ghana.

**Figure:**
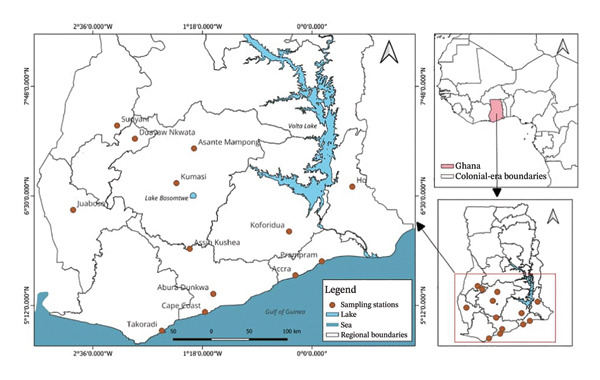
(a)

**Figure:**
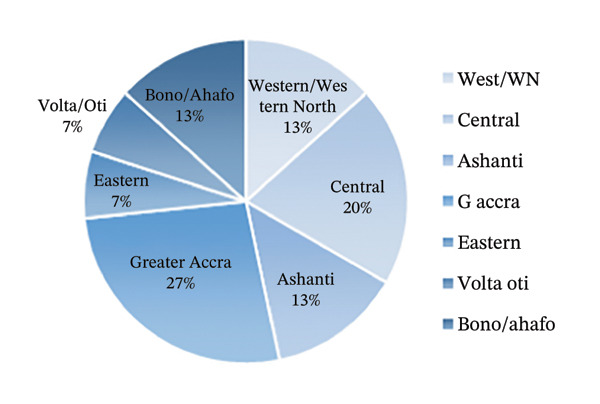
(b)

FIGURE 10(a) Comparison of the content of 5‐HMF in the analysed honey samples using student’s *t*‐test. Results are presented as means ± standard deviation (*p* value = 0.620, 95% CI) (grouped means: farm = 8.334 ± 5.291; wild = 11.10 ± 10.09), and (b) comparison of the content of 5‐HMF in the analysed honey samples using student’s *t*‐test. Results are presented as means ± standard deviation (*p* value = 0.1154, 95% CI) (grouped means: raw = 10.76 ± 9.014; regular = 1.664 ± 0.2108).

**Figure:**
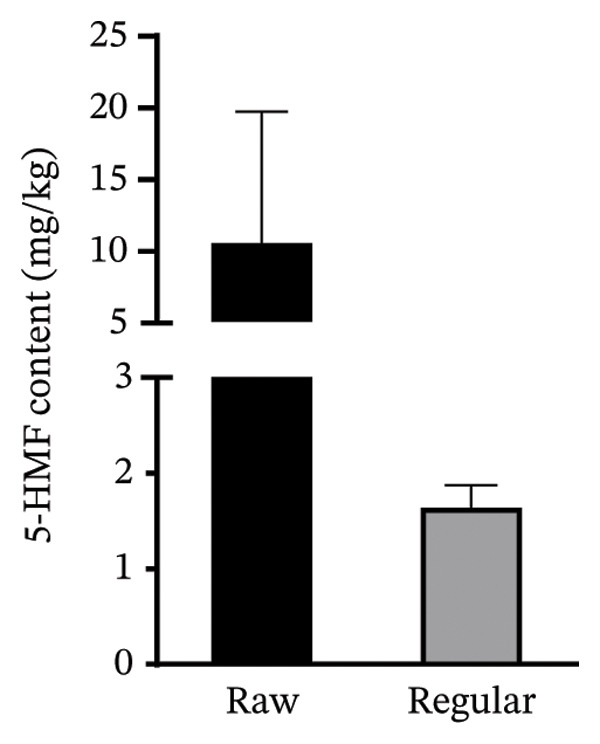
(a)

**Figure:**
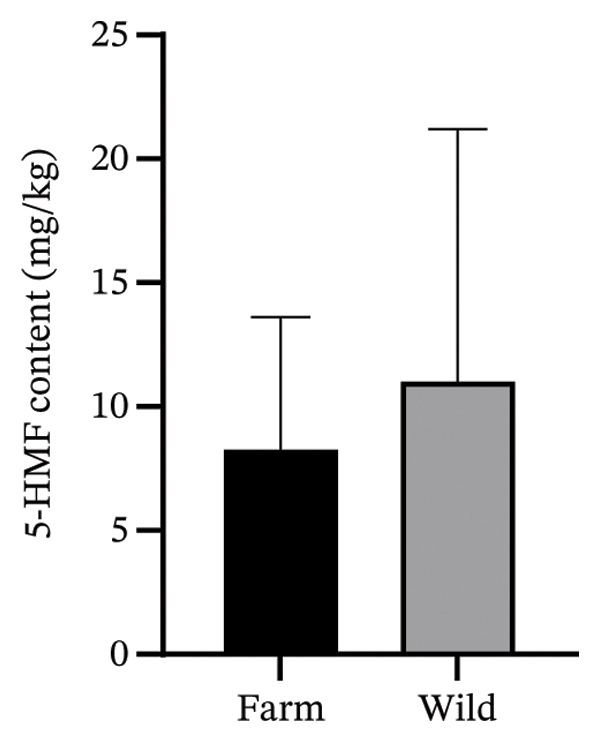
(b)

**TABLE 6:** Results from the analysis of the honey samples presented as their average content ± standard deviation.

Brand	Mean content (mg per 1 kg honey)	St. dev (±)
GBK1	81.1619	2.1169
GBV	1.1978	0.0290
GBAc1	13.9986	0.1291
GBAc2	6.3408	0.0014
GBAc3	10.9457	0.0252
GLC1	17.9179	0.4690
FB1	1.8824	0.0825
FB2	1.4618	0.0239
FB3	2.7145	0.0129
FB4	1.6467	0.0066
FB5	1.3623	0.2365
GLS1	2.2506	0.1939
GBAh	1.9837	0.4114
GBW	1.647	0.2040
GBK2	0.8176	0.1820
GBC	14.0660	0.1537
GLE	12.9635	0.1208
GLW	18.6956	0.6253
GLC	29.5852	0.1743
GLS2	2.5056	0.1407

*Note:* G, Ghanaian product; F, non‐Ghanaian product; L, local/unbranded; B, branded; A, Greater Accra; K, Ashanti; C, Central, S, Bono/Ahafo; W, Western/Western North; V, Volta/Oti; E, Eastern; c, specific brand; h, specific brand.

Eleven samples were raw honey, 3 had been taken through processing, whilst the processing state of 6 samples were unspecific.

5‐HMF was seen to be present in all the samples, with levels relatively higher in the local branded samples (mean = 14.68 mg/kg) than in the local unbranded samples (mean = 13.99 mg/kg). The foreign brands were the least of the three (1.814, *p* value = 0.4330). 5‐HMF is known to form in most foods containing sugars due to factors such as pH, period of storage, temperature and others [23].

The unprocessed raw honey samples contained less than 40 mg/kg of 5‐HMF consistent with less manipulation techniques [4].

5‐HMF levels observed in the processed honey samples (mean = 1.664 mg/kg) were relatively lower than the raw honey (mean = 10.76 mg/kg). This may be mainly due to the proportion of honey samples indicated as ‘Processed’ originating from countries like South Africa and Egypt, which are of relatively lower atmospheric temperatures compared to Ghana. Again, processing methods like pasteurisation which involves heat application over a longer period of time is able to favour the formation of 5‐HMF. However, the method employed in all 3 foreign processed brands was radurisation or irradiation, which uses ionising radiation to kill bacteria, spores and other microbes. This process has been reported by Bera et al. to reduce the amount of 5‐HMF in honey samples [25].

Both the wild and farmed honey samples contained 5‐HMF levels less than 40 mg/kg. The farmed honey types were observed to have lower mean 5‐HMF content than the wild type (*p* = 0.620).

Notably the honey sample with the highest 5‐HMF (GBK2) content was locally sourced, branded, but had no assigned FDA registration, nor date of expiry on the package. Extracting honey from honeycombs comprises a series of processes which may require heat. Considering the conditions under which 5‐HMF is formed and its use as a marker to prove the real unprocessed nature of honey, freshness of extraction or prolonged storage and high concentrations of HMF in honey points to overheating, poor storage or aged honey [12, 23, 26].

All the Ghanaian brands which were FDA‐registered honey samples contained less than 80 mg/kg of the target compound (ranging 1.647–13.9986 mg/kg) and can be said to have passed the quality assessment test for 5‐HMF in the Revised Codex Standard for Honey, 2001 [1, 20].

All the locally sampled honey passed the quality test with respect to 5‐HMF except one which contained 5‐HMF value slightly above the recommended limit of ≤ 80 mg/kg for honey originating from tropical regions of the globe [1, 19]. To be able to accurately estimate 5‐HMF at desirable levels, adopting systematic and fundamental approaches, inculcating the factors affecting its formation, is highly recommended [23].

## 4. Conclusions

An HPLC method of assaying 5‐HMF content in honey has been successfully developed. This method is fast, simple, easy to use and requires the use of cheaper solvents like distilled water and methanol. The method has been validated in accordance with the ICH guidelines and can be used to quantify 5‐HMF in different types, brands and sources of honey. The method was determined to be appropriate for assaying 5‐HMF content and produces consistent results based on the required validation parameters including robustness, reproducibility and consistency of experimental results. The method shows that the integrity of 5‐HMF in the samples was preserved over the period of analysis, verified by stability study, considering the dissolution solvent of choice, methanol. 5‐HMF content ranging from 0.8176 to 81.1619 mg per kilogram of honey was recorded from 20 honey samples, with 19 found to be within acceptable limits [1, 20]. This further highlights the quality of honey from the sampled regions and their potential for use in various products from the local pharmaceutical industry.

## Funding

This project was not funded by any external or commercial agency.

## Conflicts of Interest

The authors declare no conflicts of interest.

## Supporting Information

Supporting Table 1: Details of market samples of honey analysed.

Supporting Figure 1: Infrared spectrum of 5‐HMF reference sample used.

Supporting Figure 2: Chromatogram showing robustness at 290 nm, using 10.0 μg/mL 5‐HMF solution.

Supporting Figure 3: Chromatogram showing robustness at 297 nm, using 10.0 μg/mL 5‐HMF solution.

Supporting Figure 4: Chromatogram showing robustness at 18°C, using 10.0 μg/mL 5‐HMF solution.

Supporting Figure 5: Chromatogram showing robustness at 22°C, using 10.0 μg/mL 5‐HMF solution.

Supporting Figure 6: Chromatogram of market sample GBK1.

Supporting Figure 7: Chromatogram of market sample GBV.

Supporting Figure 8: Chromatogram of market sample GBAc2.

Supporting Figure 9: Chromatogram of market sample GBAc3.

Supporting Figure 10: Chromatogram of sample GLC1.

Supporting Figure 11: Chromatogram of market sample FB3.

Supporting Figure 12: Chromatogram of market sample FB4.

Supporting Figure 13: Chromatogram of market sample FB5.

Supporting Figure 14: Chromatogram of market sample GBAh.

Supporting Figure 15: Chromatogram of market sample GBK2.

Supporting Figure 16: Chromatogram of market sample GBC.

Supporting Figure 17: Chromatogram of market sample GLE.

Supporting Figure 18: Chromatogram of market sample GLW.

Supporting Figure 19: Chromatogram of market sample GLC2.

Supporting Figure 20: Chromatogram of market sample GLS2.

## Supporting information


**Supporting Information** Additional supporting information can be found online in the Supporting Information section.

## Data Availability

Data from this study can be made available by the corresponding author upon reasonable request.
