# Supplementary material for: Determination of 5‐Hydroxymethylfurfural Content in Marketed Honey: A Modified RP‐HPLC Method
Source: Int J Anal Chem. 2026 Apr 21;2026:9780039. doi: 10.1155/ianc/9780039 (PMC13096723; doi:10.1155/ianc/9780039)
Supplement: Supplementary file 1 — Supporting Information Additional supporting information can be found online in the Supporting Information section. [file IANC-2026-9780039-s001.docx]

**DETERMINATION OF 5-HYDROXYMETHYLFURFURAL CONTENT IN MARKETED ‘NATURAL HONEY’: AN ALTERNATIVE CHROMATOGRAPHIC TECHNIQUE**

Barbara Adu-Brimpong^1a*^, Abena Amponsaa Brobbey^2b^, Joseph Kwasi Adu^2c^, John Nii Addotey^2d^, Mustapha Kobina Abeka^1e^, Isaac Yaw Attah^1f^

^1^Department of Pharmaceutical Chemistry, School of Pharmacy and Pharmaceutical Sciences, University of Cape Coast (UCC), Cape Coast, Ghana

^2^Department of Pharmaceutical Chemistry, Faculty of Pharmacy and Pharmaceutical Sciences, Kwame Nkrumah University of Science and Technology (KNUST), Kumasi, Ghana

*[Corresponding author]

[b.adubrimpong@ucc.edu.gh](mailto:b.adubrimpong@ucc.edu.gh)^a^

[aabrobbey@knust.edu.gh](mailto:aabrobbey@knust.edu.gh)^b^

[jadu.pharm@knust.edu.gh](mailto:jadu.pharm@knust.edu.gh)^c^

[jnaddotey.pharm@knust.edu.gh](mailto:jnaddotey.pharm@knust.edu.gh)^d^

[mustapha.abeka@ucc.edu.gh](mailto:mustapha.abeka@ucc.edu.gh)^e^

[isaac.attah@ucc.edu.gh](mailto:isaac.attah@ucc.edu.gh)^f^

SUPPLEMENTARY DATA

## Supplementary table 1: Details of market samples of honey analysed

| Description, Packaging | CODE NAME | BATCH NUMBER | SOURCE | PROD. & EXPIRY DATE | F.D.A. REGISTRATION  ON PACKAGE |
| --- | --- | --- | --- | --- | --- |
| 1. Branded   Plastic bottle, 500ml | GBK1 | Unavailable | Ashanti, Ghana | Unavailable | Unavailable |
| 1. Branded   Plastic bottle, 330ml | GBV | LPH02 | Volta/Oti, Ghana | Apr. 2022- Apr. 2025 | YES |
| 1. Branded   Glass jar, 630g | GBAc1 | 21DWJ356 | Greater Accra, Ghana | Feb. 2022- Feb. 2025 | YES |
| 1. Branded   Plastic bottle, 450g | GBAc2 | 21NA1101 | Greater Accra, Ghana | Feb. 2022- Feb. 2025 | YES |
| 1. Branded   Plastic bottle, 450g | GBAc3 | 21SE742 | Greater Accra, Ghana | Mar. 2022- Mar. 2025 | YES |
| 1. Local Unbranded, 400ml | GLC | Unavailable | Central Region, Ghana | NOT INDICATED | Unavailable |
| 1. Branded   Plastic bottle, 340g (Irradiated) | FB1 | Unavailable | South Africa, Ghana | Feb. 2022- Feb. 2025 | Unavailable |
| 1. Branded   Plastic bottle, 375g (Irradiated) | FB2 | H1380A | South Africa, Ghana | Oct. 2021- Apr. 2023 | Unavailable |
| 1. Branded   Plastic bottle, 400g | FB3 | OXB2149 | Dubai, UAE | Aug. 2021- Jul. 2023 | Unavailable |
| 1. Branded   Plastic bottle, 375g (Radurised) | FB4 | 6156 | South African | --Nov. 2023 | Unavailable |
| 1. Branded   Plastic bottle, 500g | FB5 | Unavailable | Egypt | Nov. 2021- Oct. 2023 | Unavailable |
| 1. Local Unbranded, 600g | GLS | Unavailable | Bono/Ahafo | Unavailable | Unavailable |
| 1. Branded   Plastic bottle, 350ml | GBAh | Unavailable | Greater Accra | Jan. 2025 | YES |
| 1. Branded   Plastic bottle, 250ml | GBW | 03 | Western | Mar 2023- Jun 2026 | YES |
| 1. Branded   Plastic bottle, 250ml | GBK2 | Unavailable | Ashanti | Unavailable | Unavailable |
| 1. Branded   Plastic jar, 150ml | GBC | 055 | Central | May 2020- May 2024 | Unavailable |
| 1. Local Unbranded, 200ml | GLE1 | Unavailable | Eastern | Unavailable | Unavailable |
| 1. Local Unbranded, 100ml | GLW | Unavailable | Western | Unavailable | Unavailable |
| 1. Local Unbranded, 250ml | GLC2 | Unavailable | Central | Unavailable | Unavailable |
| 1. Local Unbranded, 100ml | GLS2 | Unavailable | Bono/Ahafo | Unavailable | Unavailable |

##
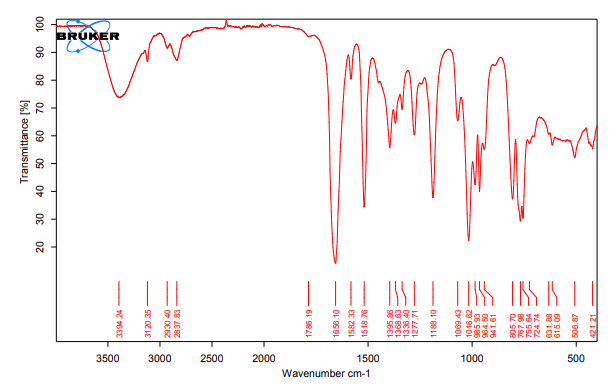


Supplementary figure 1: Infrared spectrum of 5-HMF reference sample used


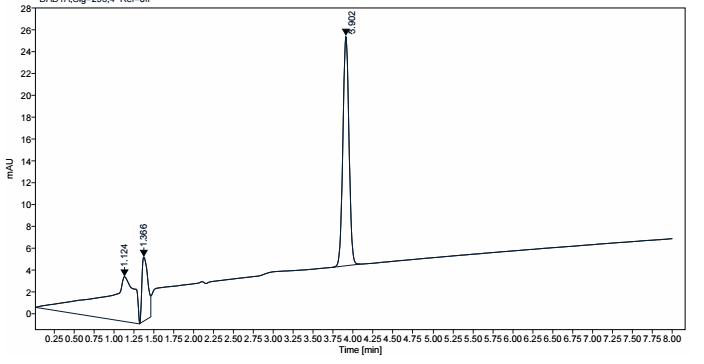
Supplementary figure 2: Chromatogram showing Robustness at 290nm, using 10.0µg/ml 5-HMF solution


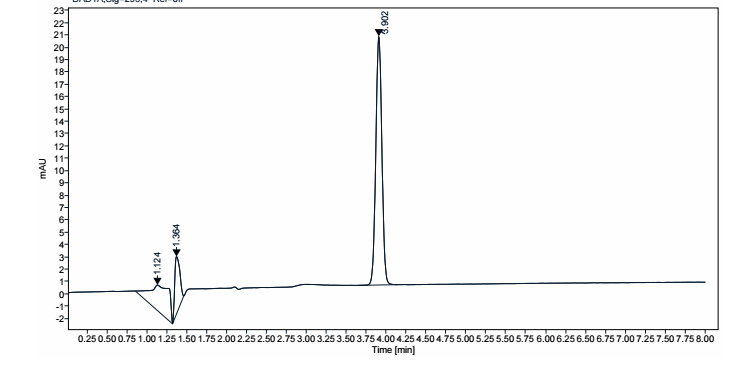
Supplementary figure 3: Chromatogram showing Robustness at 297nm, using 10.0µg/ml 5-HMF solution


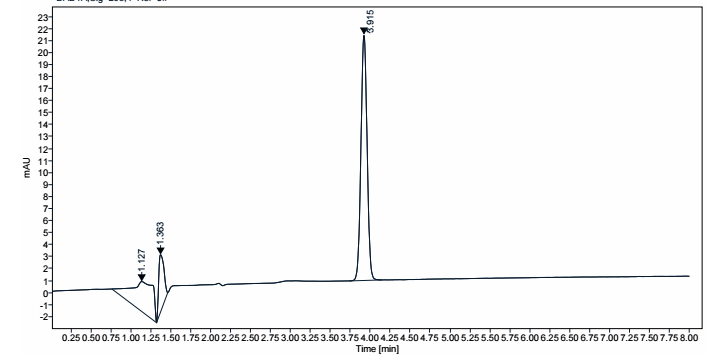
Supplementary figure 4: Chromatogram showing Robustness at 18˚C, using 10.0 µg/ml 5-HMF solution


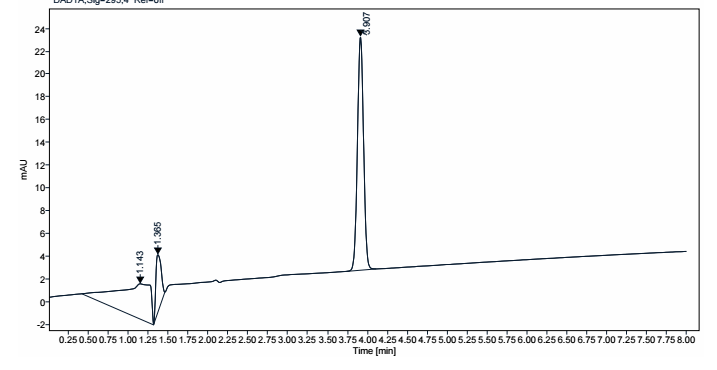
Supplementary figure 5: Chromatogram showing Robustness at 22˚C, using 10.0 µg/ml 5-HMF solution


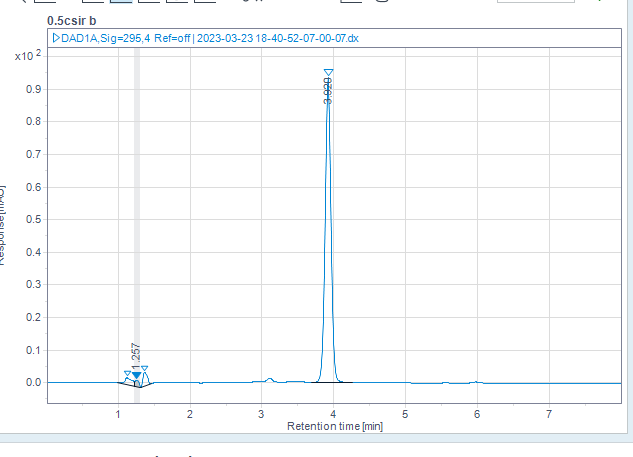
Supplementary figure 6: Chromatogram of market sample GBK1


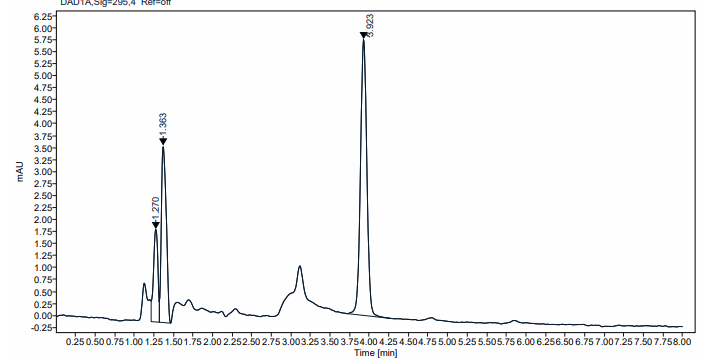
Supplementary figure 7: Chromatogram of market sample GBV


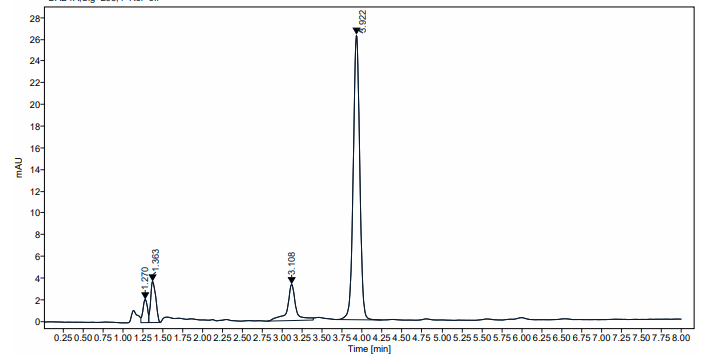
Supplementary figure 8: Chromatogram of market sample GBAc2


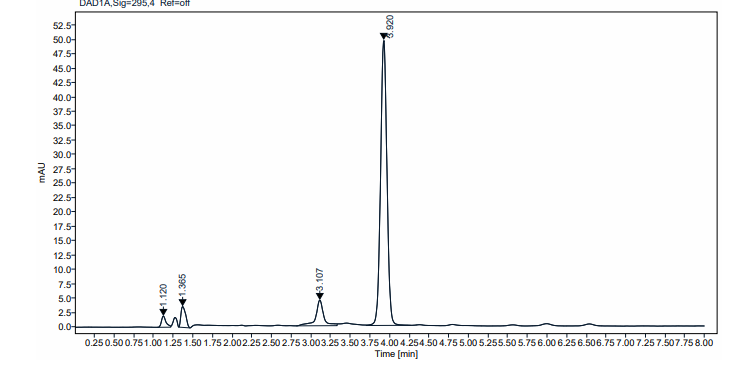
Supplementary figure 9: Chromatogram of market sample GBAc3


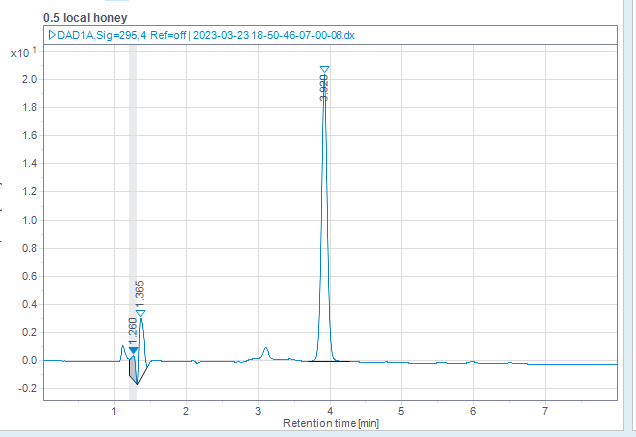
Supplementary figure 10: Chromatogram of sample GLC1


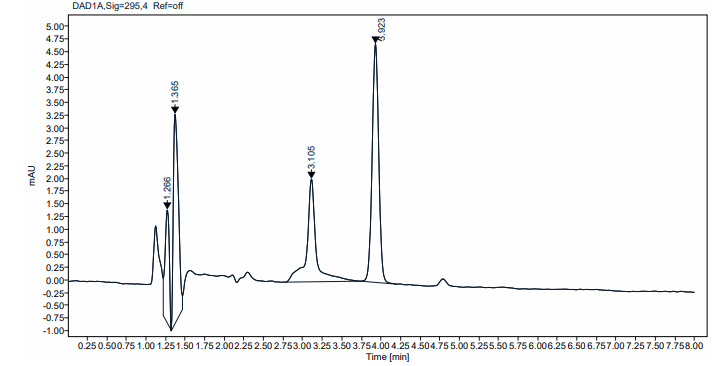
Supplementary figure 11: Chromatogram of market sample FB3


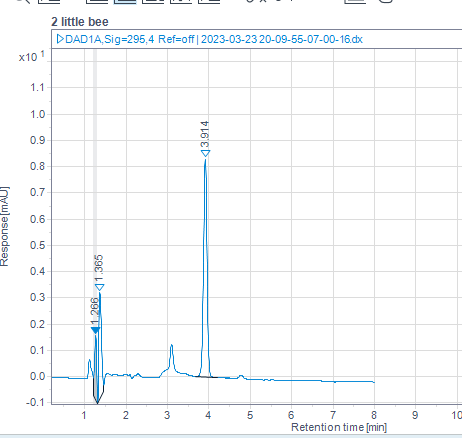


Supplementary figure 12: Chromatogram of market sample FB4


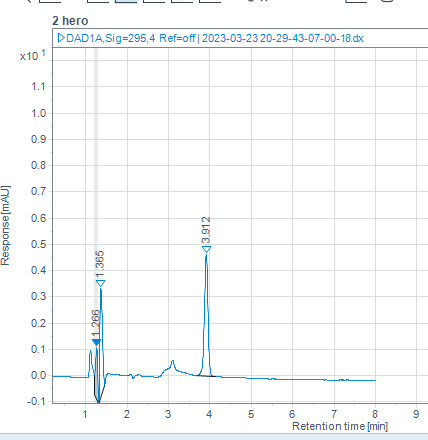


### Supplementary figure 13: Chromatogram of market sample FB5


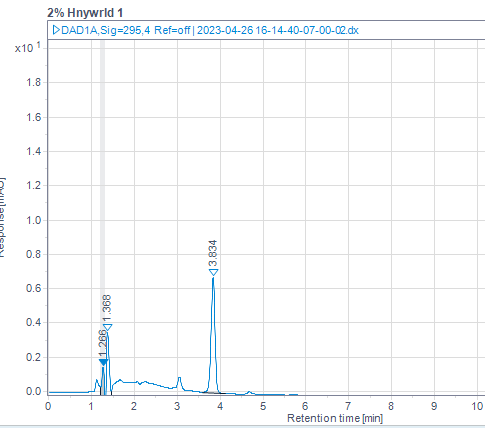


Supplementary figure 14: Chromatogram of market sample GBAh


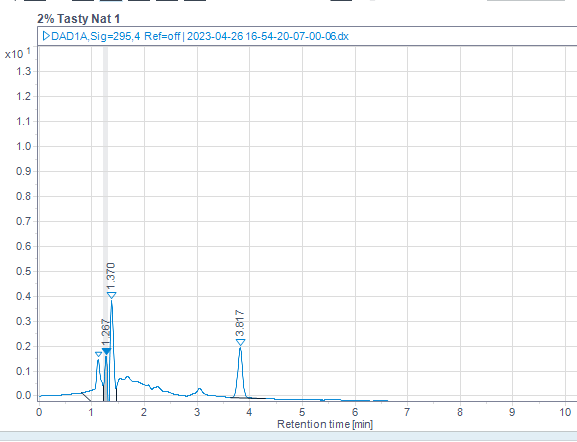
Supplementary figure 15: Chromatogram of market sample GBK2


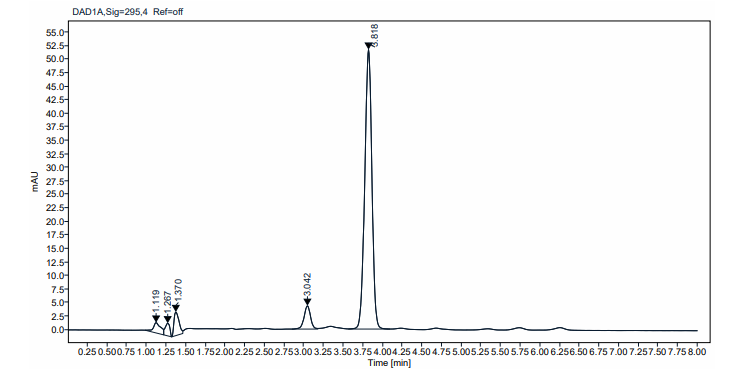
Supplementary figure 16: Chromatogram of market sample GBC


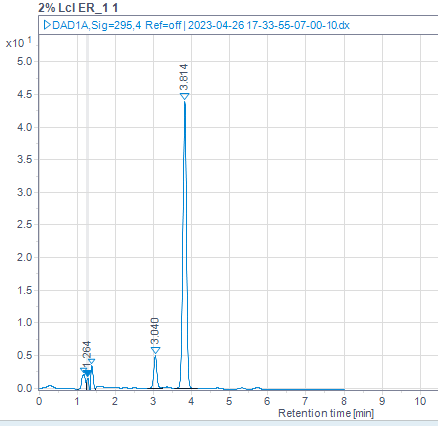


Supplementary figure 17: Chromatogram of market sample GLE


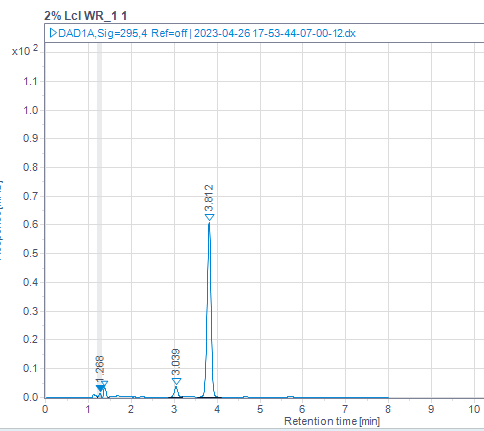


Supplementary figure 18: Chromatogram of market sample GLW


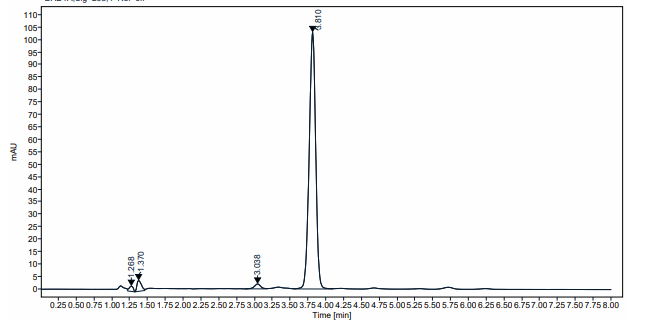


Supplementary figure 19: Chromatogram of market sample GLC2


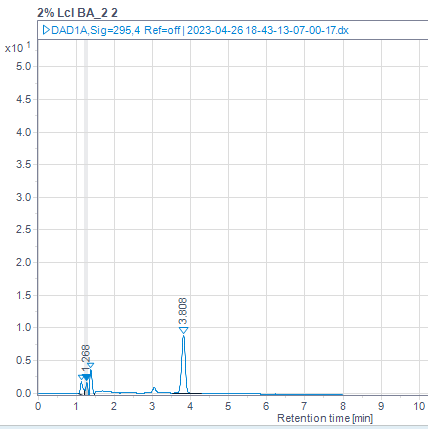


Supplementary figure 20: Chromatogram of market sample GLS2
